# Evaluating the efficacy of HRZE-based regimens in a high-burden murine model: a back-translational assessment of rifamycins and moxifloxacin substitutions in tuberculosis treatment

**DOI:** 10.3389/fphar.2025.1667592

**Published:** 2025-09-15

**Authors:** Jason E. Cummings, Lisa K. Woolhiser, Vincent Guglielmi, Machenzie Wernsman, Ashley Romano, Samantha Pauly, John T. Belisle, Nicholas D. Walter, Gregory T. Robertson, Richard A. Slayden

**Affiliations:** 1 Mycobacteria Research Laboratories, Microbiology, Immunology and Pathology, Colorado State University, Fort Collins, CO, United States; 2 Consortium for Applied Microbial Metrics, Aurora, CO, United States; 3 Division of Pulmonary Sciences and Critical Care Medicine, University of Colorado Anschutz Medical Campus, Aurora, CO, United States; 4 Rocky Mountain Regional VA Medical Center, Aurora, CO, United States

**Keywords:** high-burden aerosol BALB/c model, high burden, tuberculosis, mycobacteria, drug discovery

## Abstract

**Introduction:**

The standard treatment for tuberculosis is the isoniazid, rifampicin, pyrazinamide, and ethambutol (HRZE) regimen. Despite its efficacy, this regimen has limitations, including prolonged treatment duration and poor clinical outcomes in drug-resistant cases. This back translational study assessed the efficacy of alternative drug combinations, focusing on high-dose rifamycins (rifampicin and rifapentine) and substituting moxifloxacin for ethambutol in the HRZE regimen.

**Methods:**

Using a preclinical high-burden aerosol model of tuberculosis in BALB/c mice, we tested seven treatment combinations, including high-dose rifampicin (HD-RIF), high-dose rifapentine (HD-RPT), and moxifloxacin.

**Results:**

By day 12, the HD-RIF+HZM and HD-RPT+HZM regimens reduced lung bacterial burdens from 6.59 ± 0.08 log_10_ CFU in untreated controls to 3.70 ± 0.19 and 3.91 ± 0.43 log_10_ CFU, respectively. By day 54, bacterial loads were undetectable (<1 log_10_ CFU) in all groups except for HRZE (1.48 ± 0.32 log_10_ CFU). RS ratio analysis showed lower ratios for HD-RIF+HZM and HD-RPT+HZM compared to HRZE by day 26, indicating a superior ability of both regimens to interrupt rRNA synthesis. Histopathological analysis revealed similar granulomatous changes across all treatment groups. Mass spectrometry confirmed higher systemic exposure for HD-RIF and HD-RPT groups than RIF used in HRZE.

**Discussion:**

The findings indicate that higher doses of rifamycins and the substitution of moxifloxacin offer improved bactericidal activity and could shorten TB treatment duration.

## Introduction

Tuberculosis (TB) remains a major global health challenge, with approximately 10 million new cases each year, and the number has been increasing since 2021 (WHO, 2024). The HRZE regimen, consisting of isoniazid (H), rifampicin (R), pyrazinamide (Z), and ethambutol (E), has set the benchmark standard for TB treatment for drug-susceptible pulmonary tuberculosis. This regimen, introduced in the 1970s, has proven highly effective, reducing treatment duration to 6 months with a 2-month intensive phase (HRZE) followed by a 4-month continuation phase (HR) (Haigh et al., 2024; Sa et al., 2025). The HRZE regimen, though effective, has limitations warranting potential modifications, making it applicable to the contemporary clinical situation. HRZE treatment requires 6 months of adherence, and adverse effects such as hepatotoxicity can limit patient compliance (Semwal et al., 2025). Additionally, increasing rates of multidrug-resistant TB (MDR-TB) and extensively drug-resistant TB (XDR-TB) have underscored the need for updated RIF-sparing therapeutic regimens that can shorten treatment duration and prevent resistance (Farhat et al., 2024). When resistance emerges, treatment duration increases to 18–24 months, with a significant rise in adverse drug reactions and treatment failure rates (Trajman et al., 2025).

There is growing interest in optimizing existing TB therapies by increasing the dosage of rifamycins and replacing components such as ethambutol with more potent alternatives such as fluoroquinolones (Boeree et al., 2017; Espinosa-Pereiro et al., 2022; Dorman et al., 2021; Conde et al., 2016; Jindani et al., 2023; Carr et al., 2022). High-dose rifamycins, particularly rifampicin and rifapentine, have improved bactericidal activity and reduced treatment duration in animal models and clinical settings (Boeree et al., 2017; Boeree et al., 2015; Zhang et al., 2022; Espinosa-Pereiro et al., 2025). Rifapentine, which has a longer half-life and higher potency, potentially allows for less frequent dosing and reduces the risk of resistance (Carr et al., 2022). Moxifloxacin, a fluoroquinolone with excellent activity against *Mycobacterium tuberculosis*, has demonstrated superior sterilizing activity, particularly in regimens designed to treat MDR-TB (Dorman et al., 2021; Carr et al., 2022; Naidoo et al., 2017). The replacement of ethambutol, a mycobacterial cell wall inhibitor, with moxifloxacin, a bacterial DNA gyrase inhibitor, has shown promise in improving the bactericidal activity of the HRZE regimen while potentially reducing the development of drug resistance (Budak et al., 2023). Moxifloxacin has demonstrated superior sterilizing activity in preclinical studies and has been evaluated in clinical trials such as the REMoxTB study (NCT00864383) (Li et al., 2015; ClinicalTrials.gov, 2017; Walter et al., 2021). The trial demonstrated that substituting moxifloxacin for ethambutol in the HRZE regimen could achieve similar or better treatment outcomes in shorter periods, although it did not ultimately reduce treatment duration (Gillespie et al., 2014). Unlike these earlier efforts, the more recent Tuberculosis Trials Consortium (TBTC) Study 31/A5349 trial (NCT02410772) demonstrated that a 4-month regimen of high-dose rifapentine, isoniazid, pyrazinamide, and moxifloxacin (HPZM) was non-inferior to the standard 6-month HRZE regimen. This breakthrough represents a major milestone in TB therapy (Dorman et al., 2021; ClinicalTrials.gov, 2024).

Despite these advances, drug resistance remains a critical obstacle in TB control. Multiple factors, including suboptimal dosing, poor patient adherence, and inadequate drug exposure in infected tissues, influence the development of resistance (Farhat et al., 2024). High-dose rifamycins, such as rifampicin and rifapentine, have shown promise in overcoming some of these barriers, but have not been fully evaluated through translational studies in preclinical animal models (Arbiv et al., 2022). Studies have also indicated that increasing the dose of these drugs can enhance their bactericidal activity, reduce bacterial burden more rapidly, and potentially shorten treatment duration (Arbiv et al., 2022). By achieving higher drug concentrations in infected tissues, high-dose rifamycins may reduce the likelihood of the emergence of resistant strains, which is a critical goal in TB treatment optimization.

In this context, the goal of this study was to back-translate “Rifapentine-containing treatment shortening regimens” (TBTC Study 31/A5349) to a preclinical murine model. Specifically, we examined the bactericidal efficacy, pharmacokinetics, and pathology associated with high-dose rifamycin-based regimens containing moxifloxacin, closely mirroring the HPZM regimen evaluated in TBTC Study 31/A5349. We hypothesized that these modifications would enhance bactericidal activity and reduce the bacterial burden and pathological response while maintaining acceptable safety profiles. To test this hypothesis, we employed a preclinical high-burden aerosol respiratory subacute model of tuberculosis in BALB/c mice, a standard model used for TB drug efficacy studies. The specific objectives of this study were to compare the efficacy of high-dose rifampicin (HD-RIF) and high-dose rifapentine (HD-RPT) regimens to the standard HRZE regimen, assess the impact of moxifloxacin substitution for ethambutol in the HRZE regimen, and evaluate the overall impact of these regimens on bacterial burden and metabolic activity using an exploratory pharmacodynamic (PD) marker [RS ratio] that correlates with treatment-shortening activity (Walter et al., 2021; Dide-Agossou et al., 2022), pathology, and treatment tolerability. By assessing bacterial reduction, lung pathology, drug pharmacokinetics, and treatment tolerability, we determined that these regimens outperformed the standard HRZE regimen and offered alternatives for shortening TB treatment duration while maintaining efficacy. This study builds on previous research and seeks to contribute to developing more effective and shorter treatment regimens for TB, potentially leading to improved patient outcomes and reduced disease transmission.

## Methods

### Study design and animal model

**FIGURE 1:**
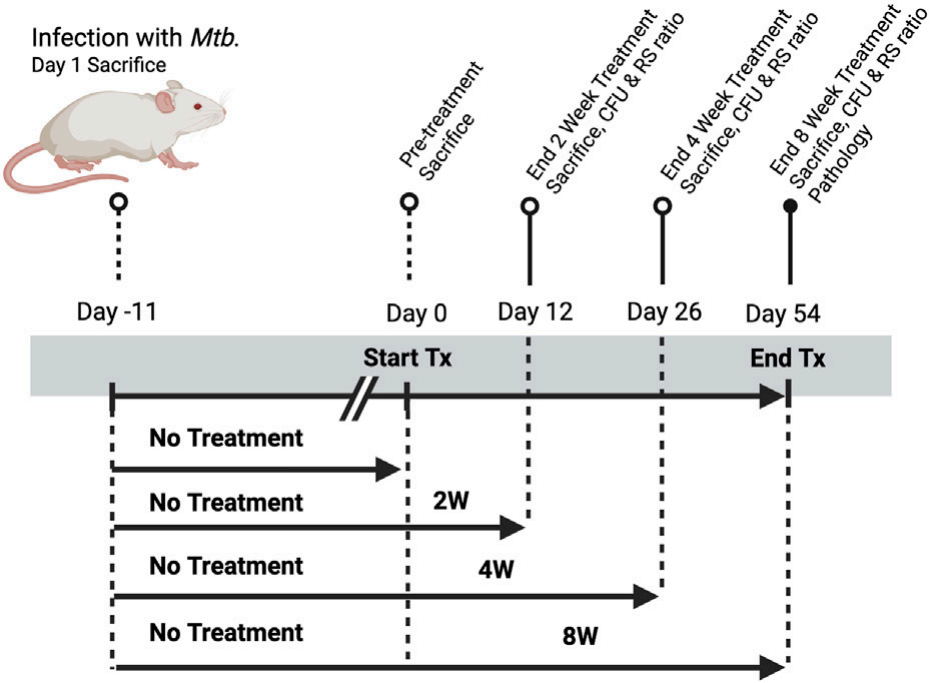
Study design for evaluating treatment duration-dependent responses to standard HRZE therapy in a murine model of *Mycobacterium tuberculosis* infection. Mice were aerosol-infected with *M. tuberculosis* on Day–11 and one group was sacrificed at Day 1 post-infection to establish baseline lung bacterial burden. All remaining animals remained untreated until Day 0, at which point treatment (Tx) was initiated. Groups received treatment for 2, 4, or 8 weeks, with corresponding sacrifices on Days 12, 26, and 54 to assess treatment efficacy. Endpoints included colony-forming unit (CFU) enumeration and determination of RS Ratio. Day 54 mice were also evaluated for histopathological changes and PK analysis. A parallel untreated control group was maintained through the full 8-week period.

This study evaluated the efficacy of high-dose rifamycins and the substitution of moxifloxacin in the HRZE regimen using the well-established high-burden mouse model of *M. tuberculosis* infection used in treatment evaluation (Figure 1). Female BALB/c mice, 6–8 weeks old, were aerosol-infected with the *M. tuberculosis* Erdman strain at a target of 4 log_10_ CFU in the lungs on day one post-infection. Infection was initiated 11 days before the start of treatment, and mice were sacrificed at three key time points: after 2 weeks (D12), 4 weeks (D26), and 8 weeks (D54) of treatment. Bacterial burden in the lungs was quantified at each time point. Mice were sacrificed by CO_2_ asphyxiation, and their lungs were harvested for CFU enumeration, RS ratio evaluation, and histopathological analysis 1-day following the last day of treatment. Drug concentrations were determined by mass spectrometry analysis of plasma collected at 1 h post-delivery (C_max_) and 24 h post-delivery (C_min_) during the final week of treatment. Each group contained 10 animals per time point, with 4 additional animals included for histopathology at 8W TP. Only 1 animal (mouse E; HDRPT + HZE, 8W TP) was excluded from analysis due to mortality following blood collection; there were no other instances of animal loss requiring handling of missing data. This single animal exclusion did not impact the data structure, distribution, or statistics. All remaining mice tolerated dosing well, and weight monitoring and clinical observations did not reveal adverse effects attributable to treatment.

The study tested seven treatment regimens, including the standard HRZE regimen and its modified forms with high-dose rifamycins and moxifloxacin substitutions. The drugs tested were isoniazid (10 mg/kg), rifampicin (10 mg/kg), high-dose rifampicin (HD-RIF; 30 mg/kg), pyrazinamide (150 mg/kg), ethambutol (100 mg/kg), high-dose rifapentine (HD-RPT; 20 mg/kg), and moxifloxacin (100 mg/kg). Treatments were administered via oral gavage 5 days per week for two, four, and 8 weeks. Drug formulations were prepared weekly, and doses were adjusted based on cage weight averages to ensure accurate drug delivery. The treatment groups were as follows: Untreated control group, HRZE regimen (isoniazid, rifampicin, pyrazinamide, ethambutol), High-dose rifampicin alone, High-dose rifampicin combined with HZE, High-dose rifampicin combined with HZ and moxifloxacin, High-dose rifapentine alone, High-dose rifapentine combined with HZE, and High-dose rifapentine combined with HZ and moxifloxacin.

### Determination of bacterial burden and RS ratio

CFU counts were determined by serial dilution of lung homogenates on 7H11 agar plates, and colonies were allowed to grow for up to 5 weeks. Results were reported as log_10_ CFU per lung. The lower limit of detection for the CFU assay was approximately 1.0 log_10_ CFU per lung. RS ratio analysis was conducted to provide an additional measure of the efficacy of the tested drug regimens. The RS ratio is a highly precise exploratory pharmacodynamic measure that quantifies ongoing *M. tuberculosis* rRNA synthesis and is a measure of a drug regimen’s effect on relative bacterial metabolic activity. A reduction in RS ratio between treatment time points and the start of treatment has been shown to correlate with regimen treatment-shortening activity (Walter et al., 2021; Dide-Agossou et al., 2022). This analysis provides a more sensitive measure of the drug’s effect, particularly at later time points when CFU counts approach the lower detection limit. RS ratio values were calculated as described previously (Walter et al., 2021). A lower RS ratio indicates a more significant reduction in bacterial activity relative to the pre-treatment level and, thus, a more effective drug regimen. RS ratio analysis was used to further stratify the tested regimens’ efficacy, especially for differentiating between high-dose rifamycins and standard HRZE regimens.

### Clinical observations and histopathology

Lung tissues were subjected to histopathological analysis to evaluate the extent of granulomatous inflammation and other pathological changes associated with the treatments. Histopathology was performed on the lungs of select mice at the pre-treatment and day 54 time points. Hematoxylin and eosin (H&E) staining was used to assess the lung tissue for granulomas, lymphocytic infiltration, and the presence of inducible Bronchus-Associated Lymphoid Tissue (iBALT). The formation of iBALT was used as a marker of the lung’s immune response to *M. tuberculosis* infection and drug treatments. Body weight was monitored throughout the study as a general measure of health and treatment tolerance. Mice were weighed weekly, and changes in body weight were recorded to assess the potential toxicity of the different drug regimens (Supplementary Figure S1).

Lung perfusions were performed after clamping the caudal and cranial vena cava to retain 4% paraformaldehyde (PFA) in the lungs. An incision was made into the left ventricle of the heart for blood to drain, and a syringe with 26G 1/2 inch needle was used to inject 10 mL of 4% PFA into the right ventricle for lung fixation. Lungs were then placed into histology cassettes and fixed submerged in 4% PFA. After 48 h, lungs were placed into fresh 1xPBS. Whole lungs were sectioned and stained with H&E. A board-certified veterinary anatomic pathologist, who was partially blinded to the treatment group assignments, performed the initial slide review. Histopathological analysis was performed on five transverse lung sections (one section from each lung lobe) using lung-specific scoring parameters (Robertson et al., 2021). Lesions were semi-quantitatively scored for each of the five lung tissue sections. Anatomic lesion categories assessed in this scheme included the percent of tissue affected, cellular composition of inflammatory foci, degree of destructive pathology, and perivascular/peribronchiolar cellular infiltrate.

### Mass spectrometry analysis

Mass spectrometry was conducted on plasma samples to determine drug levels in plasma 1-h and 24-h post administration of the last drug treatment to assess drug exposures and pharmacokinetics. Blood was collected from the mice at both time points after administering the drugs. Plasma samples were separated by centrifugation at 3,000×g for 2 min and stored at −80°C until analysis. Drug concentrations were quantified using a validated liquid chromatography-tandem mass spectrometry (LC-MS/MS) method. Rifampicin, rifapentine, pyrazinamide, isoniazid, ethambutol, and moxifloxacin were separated on a C18 column and detected with mass spectrometry. Calibration curves for each drug were prepared in plasma with known concentrations, and drug concentrations in the study samples were interpolated from these curves. The LC-MS/MS parameters were optimized for each drug based on previously published methods and included the following transitions: rifampicin (m/z 823 → m/z 791), rifapentine (m/z 847 → m/z 815), pyrazinamide (m/z 124 → m/z 81), isoniazid (m/z 138 → m/z 121), ethambutol (m/z 205 → m/z 116), and moxifloxacin (m/z 402 → m/z 384). The lower limit of quantification for each drug was 50 ng/mL. Plasma drug concentrations were analyzed to determine peak (C_max_) and trough (C_min_) levels for each drug.

### Statistical analysis

Statistical comparisons between treatment groups’ mean CFU counts and RS Ratio values were performed using ANOVA followed by *post hoc* Tukey’s multiple comparison tests to determine the significance of differences. The primary outcomes were reduced lung bacterial burden over time, reduced RS ratio values, and histopathology scores. Statistical significance was defined as p < 0.05. Data variability within groups was calculated and reported as the standard error of the mean. Quartile ranking and Cohen’s d were used to determine efficacy benchmarks. Quartile ranking categorizes treatment outcomes into performance tiers based on statistical distribution. The first quartile (Q1 or 25th percentile reduction) is the cutoff for the lowest performing treatments, and the third quartile (Q3 or 75th percentile reduction) establishes the cutoff for the highest performing therapies. Cohen’s d quantifies the effect size by comparing treatment outcomes normalized by the pooled standard deviation (Cummings and Slayden, unpublished).

## Results

### High-dose rifamycins and moxifloxacin as a substitute for ethambutol enhance bacterial clearance

**FIGURE 2:**
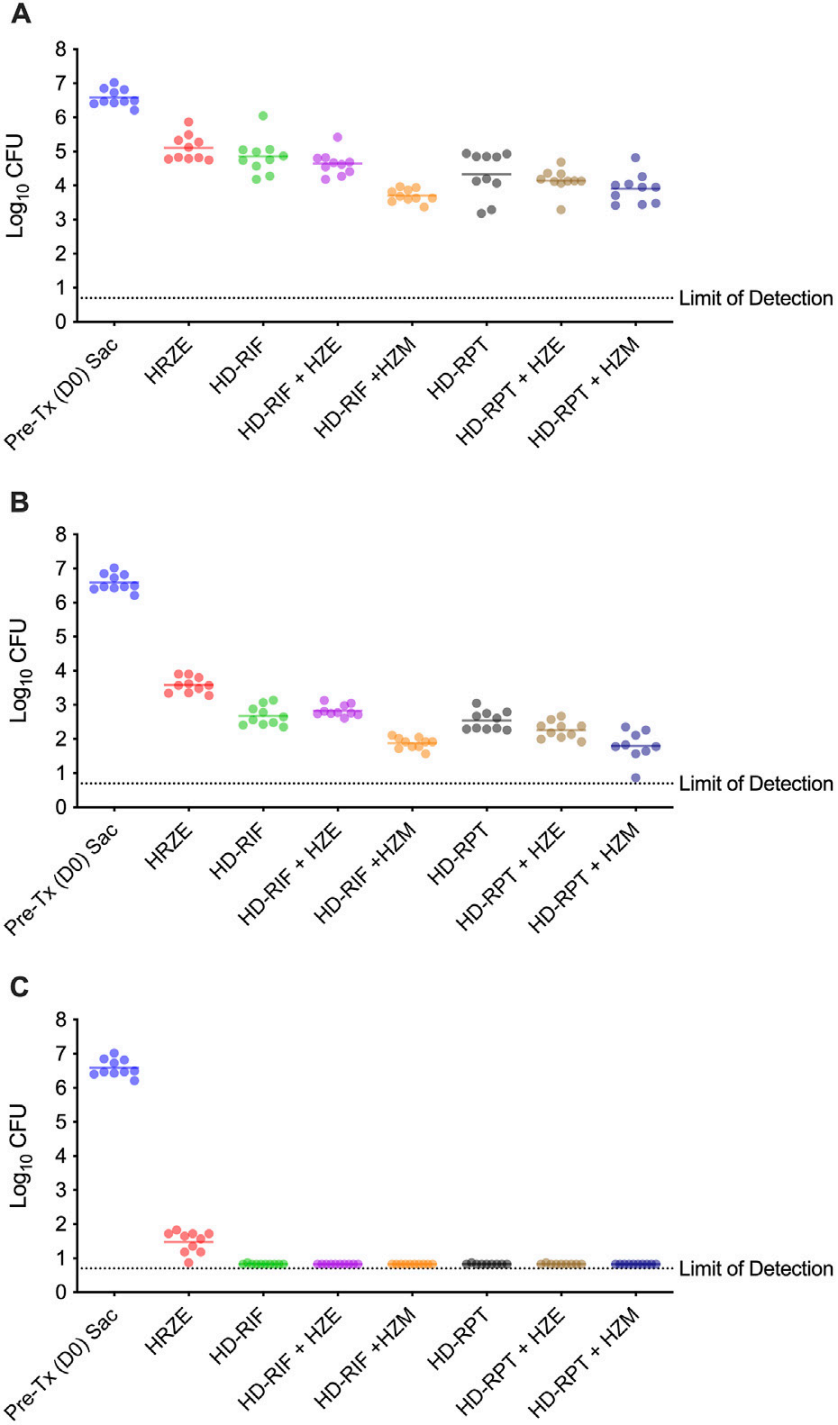
Lung bacterial burden following 2, 4, and 8 weeks of treatment. Lung colony-forming units (CFU) were quantified from mice infected with *Mycobacterium tuberculosis* and treated for 2, 4, or 8 weeks, as indicated. Individual and mean lung CFU values from pre-treated (Day 0), and each treatment at **(A)** 2-week (Day 12) **(B)** 4-week (Day 26), and **(C)** 8-week (Day 54) treatment groups. Each symbol represents an individual mouse; horizontal bars indicate group means. CFU values were log_10_-transformed for analysis. Statistical significance was determined using one-way ANOVA with *post hoc* Tukey’s multiple comparisons; *p* < 0.05 was considered significant.

The efficacy of each treatment regimen was measured by quantifying the bacterial burden in the lungs on days 12, 26, and 54 (Figure 2). At day 12, the bacterial load in the lungs of untreated control mice was 6.59 ± 0.08 log_10_ CFU, indicating robust infection. The HRZE regimen reduced the lung bacterial burden to 5.11 ± 0.12 log_10_ CFU (Mean Diff. 1.48 log_10_ CFUs; p < 0.0001). In comparison, HD-RIF alone reduced the bacterial load to 4.86 ± 0.16 log_10_ CFU (Mean Diff. 1.73 log_10_ CFUs; p < 0.0001), and HD-RPT alone reduced it to 4.33 ± 0.21 log_10_ CFU (Mean Diff. 2.26 log_10_ CFUs; p < 0.0001). The most significant reductions were observed in the HD-RIF + HZM and HD-RPT + HZM groups, with bacterial burdens of 3.70 ± 0.06 log_10_ CFU (Mean Diff. 2.89 log_10_ CFUs; p < 0.0001) and 3.91 ± 0.14 log_10_ CFU (Mean Diff. 2.68 log_10_ CFUs; p < 0.0001), respectively, representing reductions of nearly 3 log_10_ CFU compared to the untreated control (Figure 2A). By day 26, further reductions in bacterial burden were noted across all groups. Treatment with the HRZE regimen resulted in a bacterial load of 3.58 ± 0.07 log_10_ CFU (Mean Diff. 3.01 log_10_ CFUs; p < 0.0001), while HD-RIF alone reduced it to 2.68 ± 0.09 log_10_ CFU (Mean Diff. 3.90 log_10_ CFUs; p < 0.0001) and HD-RPT alone reduced it to 2.53 ± 0.09 log_10_ CFU (Mean Diff. 4.06 log_10_ CFUs; p < 0.0001). The HD-RIF + HZM and HD-RPT + HZM groups again demonstrated superior efficacy, with bacterial burdens of 1.88 ± 0.05 log_10_ CFU (Mean Diff. 4.71 log_10_ CFUs; p < 0.0001) and 1.80 ± 0.15 log_10_ CFU (Mean Diff. 4.79 log_10_ CFUs; p < 0.0001), respectively (Figure 2B). These reductions represent more than a 4.7 log_10_ CFU decrease compared to the initial bacterial burden in the lungs. At day 54, bacterial burdens in the lungs had reached the lower limit of detection (∼1.0 log_10_ CFU) for all groups except HRZE, which retained detectable bacterial levels (1.48 ± 0.10 log_10_ CFU) (Mean Diff. 5.11 log_10_ CFUs; p < 0.0001) (Figure 2C). HD-RIF + HZM and HD-RPT + HZM regimens reduced the bacterial burden in the lungs to <0.66 log_10_ CFU, with no CFU returned for any of the animals in these study groups. Statistical data for bacterial burden and effect size can be found in Supplementary Table S1. We assessed treatment performance using quartile ranking criteria of the bacterial burden reduction at each time point (Table 1). At day 12, treatments that achieved bacterial burden reductions that exceeded the upper threshold (Q4 >75%) of 4.32 log_10_ CFU were determined to be highly effective. Reductions below the 1^st^ quartile (25th percentile or Q1), or a <1.44 log_10_ CFU reduction, were ineffective (Cummings and Slayden, unpublished). On day 12, the quartile analysis revealed a lower threshold (Q1) of 1.61 log_10_ CFU and an upper threshold (Q3) of 2.56 log_10_ CFU. HD-RIF + HZM and HD-RPT + HZM were classified as highly effective, and HRZE was classified as having little to no efficacy observed by day 12, and all other regimens were minimally to moderately effective. By day 26, the Q1 threshold increased to 3.35 log_10_ CFU and Q3 to 4.48 log_10_ CFU, although this benchmark increase did not change the results compared to what was observed on day 12. Lastly, on day 54, the Q1 threshold increased to 5.72 log_10_ CFU and Q3 to 5.76 log_10_ CFU. All treatments besides HRZE were classified as highly effective by day 54. To determine the significance of these thresholds, we used effect size ranking using Cohen’s d for all treatment groups compared to pre-treatment sacrifice levels in the lung. Effect sizes were classified as minimal, moderate, large, or very large based on the quartile ranking of the Cohen’s d value dataset. For the lungs, the thresholds were minimal (<4.52), moderate (4.52–7.76), large (7.76–15.22), and very large (>15.22). By day 12, only HD-RIF + HZM and HD-RPT + HZE exhibited large effect sizes, HD-RIF and HD-RPT showed minimal effect sizes, and the remaining treatments showed moderate effect sizes. By day 26, HRZE, HD-RIF, and HD-RPT + HZM demonstrated a large effect size and all remaining groups a very large effect size. By day 54, all treatment groups demonstrated a very large effect size compared to pre-treatment.

**TABLE 1:** Performance benchmarks - quartile ranking reduction in bacterial burden (log_10_ CFU).

	Ineffective Q1 (<25%)	Minimally effective Q2 (25%–50%)	Moderately effective Q3 (50%–75%)	Highly effective Q4 (>75%)
Day 12	0–0.72	0.72–1.45	1.45–2.17	2.17–2.89
Day 26	0–1.20	1.20–2.40	2.40–3.59	3.59–4.79
Day 54	0–1.44	1.44–2.88	2.88–4.32	4.32–5.76

### RS ratio

**FIGURE 3:**
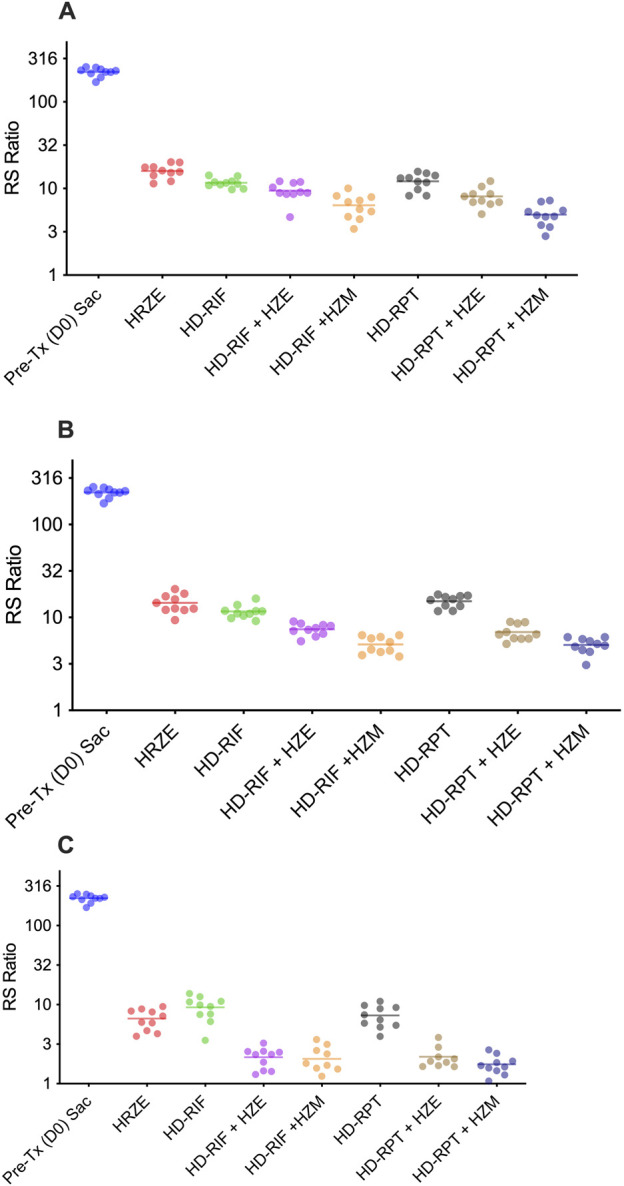
RS Ratio quantification reveals differential reductions in bacterial transcriptional activity during treatment at 2, 4, and 8 weeks. The RS Ratio was measured in lung homogenates from *Mycobacterium tuberculosis*-infected mice collected at Day 0 (pretreatment baseline), and following 2, 4, or 8 weeks of HRZE treatment (Days 12, 26, and 54, respectively). Individual and mean RS ratio values from pre-treated (Day 0), and each treatment at **(A)** 2-week (Day 12) **(B)** 4-week (Day 26), and **(C)** 8-week (Day 54) treatment groups. The RS Ratio serves as a surrogate marker for active ribosomal RNA synthesis and reflects bacterial metabolic activity. Each symbol represents an individual mouse; horizontal bars indicate group means. A progressive decrease in the RS Ratio was observed across treatment durations, consistent with cumulative suppression of transcriptional activity. Statistical comparisons were performed using one-way ANOVA followed by Tukey’s multiple comparisons test; *p* < 0.05 was considered statistically significant.

RS ratio analysis was performed as an orthogonal measure of drug regimen efficacy on a central biological process, ongoing *M. tuberculosis* rRNA synthesis, offering additional insights into the pharmacodynamics of the treatments (Figure 3). Statistical data for RS Ratio can be found in Supplementary Table S2. At day 12, the RS ratio for the HRZE regimen was 16.0, indicating a reduction in rRNA synthesis from the start of treatment. RS ratios for the high-dose rifamycin regimens were 6.42 for HD-RIF + HZM and 4.99 for HD-RPT + HZM, indicating that a rifamycin dose effect was clearly discernible by RS ratio early (Figure 3A). By day 26, the RS ratio for the HRZE regimen improved to 14.3, but this was still significantly higher than the RS ratios for HD-RIF + HZM (5.13) or HD-RPT + HZM (5.05), confirming the superior efficacy of these regimens by this pharmacodynamic measure (Figure 3B). At day 54, RS ratios in all multi-drug treatment groups dropped below 2.2, with the lowest values observed in HD-RIF + HZM and HD-RPT + HZM (2.16 and 1.41, respectively). These values indicate that continued treatment with these regimens further suppressed ongoing rRNA synthesis by RS ratio, which remained quantifiable even when CFU counts were near or below the detection limit (Figure 3C).

### Reduction in bacterial burden improved pathological outcomes

**FIGURE 4:**
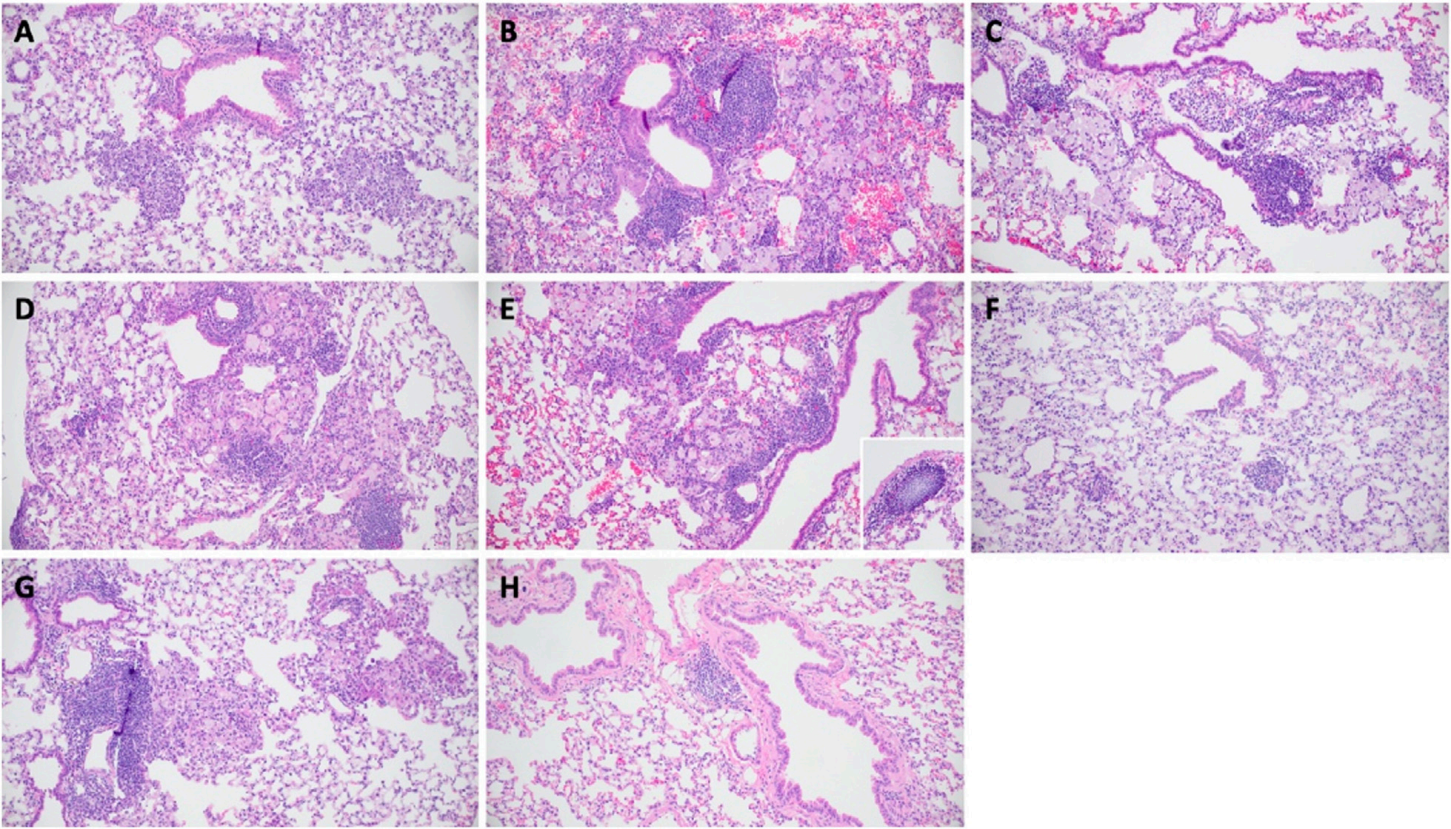
Lung histopathology of *Mycobacterium tuberculosis*-infected and treated mice. Representative hematoxylin and eosin (H&E)-stained lung tissue sections from mice infected *Mycobacterium tuberculosis* and treated. **(A)** Infected, untreated control mouse. Two distinct inflammatory foci comprised of neutrophils and histiocytes are observed. **(B)** HRZE-treated mouse showing mixed inflammatory infiltration. **(C)** HD-RIF + HZE-treated mouse with preserved alveolar structure and focal inflammation. **(D)** HD-RIF + HZM-treated mouse displaying a dense lesion with cellular infiltration. **(E)** HD-RPT + HZE-treated mouse with reduced parenchymal inflammation. Inset highlights discrete inducible bronchus-associated lymphoid tissue (iBALT); central pallor is an artifact of staining. **(F)** Another HD-PRT + HZE-treated mouse showing well-delineated inflammatory regions. **(G)** HD-RPT + HZM-treated mouse exhibiting preserved architecture with limited lesion formation. **(H)** Normal BALT structure in an uninfected, untreated control mouse, illustrating baseline pulmonary lymphoid tissue. These micrographs illustrate differential lung pathology and immune architecture remodeling induced by infection and modified by treatment.

Histopathological examination of lung tissues revealed minor inflammatory response and lesion severity differences between treatment groups. Untreated control mice showed equal numbers of foamy macrophages and neutrophils within alveolar spaces, with minimal perivascular or peribronchiolar mononuclear cell aggregates (Figure 4). On day 54, all treatment groups exhibited histopathological features that differed from those observed in untreated mice. Lungs from all treated mice demonstrated lymphohistiocytic to foamy macrophage-predominant inflammatory lesions within peribronchiolar and intra-alveolar regions with variably sized mononuclear cell aggregates expanding perivascular and peribronchiolar spaces (i.e., iBALT). Lesions in all treated groups compromised approximately 10% of lung tissue sections assessed, except for three HD-RPT + HZE mice (Figure 4). The three HD-RPT + HZE mice had notably smaller inflammatory foci composed of fewer inflammatory cells with smaller perivascular/peribronchiolar mononuclear cell aggregates (Figure 4). All HRZE-treated mice had low numbers of cholesterol clefts interspersed within several inflammatory foci. Rare cholesterol clefts and scant pyknotic to karyorrhectic debris were inconsistently observed within inflammatory foci in HD-RIF + HZE, HD-RIF + HZM, and HD-RPT + HZM treated mice.

### Treatment improved clinical signs and was well tolerated

Throughout the study, body weight was monitored to indicate general health and treatment tolerability. Mice in the HRZE and HD-RIF + HZE groups exhibited slight weight loss after the first 2 weeks of infection (approximately 3%–5% of body weight). Mice treated with the remaining regimens showed consistent weight gain, averaging a 3%–5% increase in body weight over the 8-week treatment period. No significant adverse events were reported for any of the treatment groups, indicating good tolerability of these regimens.

### High-dose rifamycins achieved greater exposure during the treatment period

Plasma concentrations of the tested drugs were measured at 1 hour (C_max_) and 24 h (C_min_) post-dose (Supplementary Table S3). High-dose rifamycins achieved greater exposure during the treatment period. Rifampicin (10 mg/kg) reached a mean C_max_ of 11,356 ng/mL and a mean C_min_ of 1,073 ng/mL. High-dose rifampicin (30 mg/kg) achieved a mean C_max_ of 28,899 ng/mL and a C_min_ of 3,942 ng/mL, confirming significantly higher systemic plasma exposures by high-dose rather than standard dosing. Rifapentine (20 mg/kg) resulted in a C_max_ of 38,024 ng/mL and a C_min_ of 29,844 ng/mL, with high-dose rifapentine also exhibiting a long half-life resulting in prolonged drug exposures compared to a standard dose of rifampicin. Moxifloxacin (100 mg/kg) reached a C_max_ of 1,051 ng/mL at 1-h post-administration but was undetectable at 24 h, consistent with its known pharmacokinetic profile. These results confirmed that the administered drug dosages achieved expected plasma exposures, with the increased exposures of rifampicin and rifapentine contributing to the improved bactericidal activity observed in the HD-RIF + HZM and HD-RPT + HZM treatment groups.

## Discussion

This study back-translates the TBTC Study 31/A5349 clinical trial regimen (HPZM) into a murine model (Espinosa-Pereiro et al., 2025). It demonstrates that high-dose rifamycins, particularly when combined with moxifloxacin, significantly improved bacterial clearance compared to the standard HRZE regimen. Both high-dose rifampicin (HD-RIF) and high-dose rifapentine (HD-RPT) regimens reduced lung bacterial loads more effectively than HRZE, with undetectable bacterial CFU levels achieved in the lungs with 2-months of treatment (day 54). These results are concordant with the RIFAQUIN clinical trial evaluating rifapentine, which determined that higher doses of rifapentine achieve greater efficacy than rifampicin in human patients with pulmonary TB (Jindani et al., 2014; Dorman et al., 2012). Similarly, another study supported the safety and efficacy of high-dose rifampicin in patients with pulmonary TB, showing that doses up to 35 mg/kg were well-tolerated and led to more rapid sputum conversion than the standard dose (Garcia-Prats et al., 2021). This result is particularly important because faster sputum conversion correlates with reduced transmission and better clinical outcomes (Svensson et al., 2018).

Further, our back translational study shows that combining moxifloxacin with high-dose rifampicin or rifapentine provides an additive treatment effect. Fluoroquinolones, particularly moxifloxacin, have long been recognized for their potent activity against *M. tuberculosis*, which is attributed to their ability to inhibit DNA gyrase, a critical enzyme in bacterial replication. Our observation is consistent with studies that showed that replacing ethambutol with moxifloxacin in the HRZE regimen reduced the time to culture conversion in patients with drug-susceptible TB (Arbiv et al., 2022). The REMoxTB trial investigated the substitution of moxifloxacin in place of ethambutol or isoniazid in the standard HRZE regimen and demonstrated that moxifloxacin-containing regimens performed similarly to HRZE (Gillespie et al., 2014). The efficacious enhancement between moxifloxacin and high-dose rifamycins observed in our study echoes the regimen structure validated in TBTC Study 31/A5349, underscoring its translational robustness (Arbiv et al., 2022). Fluoroquinolones are also a cornerstone of multidrug-resistant TB (MDR-TB) treatment regimens, as outlined by the World Health Organization (WHO), and their use in drug-susceptible TB treatment could prevent the emergence of resistance to first-line drugs. This suggests that this combination may provide a viable strategy to shorten TB treatment duration with lesser concern about TB relapse, particularly in drug-resistant cases. This study highlights the potential of fluoroquinolone-based regimens in future treatment protocols, particularly when coupled with high-dose rifamycins, to improve overall regimen potency. This is consistent with high-dose regimens, which have been evaluated in clinical studies (rifampicin ≥30 mg/kg), have been associated with low-grade adverse events (Espinosa-Pereiro et al., 2022; Dorman et al., 2021). Similarly, moxifloxacin at elevated exposures may increase the risk of QT interval prolongation and, as noted in preclinical models, may induce reactive oxygen species (ROS) generation, potentially contributing to cellular injury. The REMoxTB, PanACEA MAMS-TB-01, and TBTC Study 31/A5349 did not report significant adverse effects, which is consistent with our backtranslational findings that should therefore be interpreted as preclinical proof-of-concept data supporting regimen design principles rather than direct clinical dose recommendations.

RS ratio was used in our study as an orthogonal pharmacodynamic measure to CFU reduction and provided important insights into the antimicrobial properties of the tested regimens. RS ratio analysis showed that the HD-RIF + HZM and HD-RPT + HZM regimens remained superior to HRZE at day 54, even when CFU counts were near or below the detection limit. This suggests that the RS ratio may be a more sensitive indicator of drug efficacy, especially at later stages of treatment. It has been shown that the RS ratio is an early predictor of treatment success, with lower RS ratios correlated with reduced relapse rates and better long-term outcomes in preclinical TB models (Reichlen et al., 2023). Our study found that the RS ratios for high-dose rifamycins and moxifloxacin regimens were lower than those for HRZE, consistent with the observed improved treatment performance for high-rifamycin moxifloxacin regimens. This approach provides a bacterial metabolic metric enabling the identification of regimens that reduce ongoing rRNA synthesis, a fundamental biological metric of bacterial metabolic activity. This RS ratio metric differentiates between regimens that may otherwise appear similar based on CFU counts alone, providing a more nuanced assessment of long-term drug regimen efficacy (Dide-Agossou et al., 2022).

While our murine results demonstrate superior bactericidal activity with high-dose rifampicin and rifapentine combined with moxifloxacin, they cannot be directly extrapolated to human clinical practice. Murine pharmacokinetics and drug tolerance profiles differ substantially from humans. A recent meta-analysis of severe adverse events comparing the standard dose of rifampicin to double and triple doses treatments reported that triple dose rifampicin regimens resulted in slight increase in reported adverse effects with no deaths compared to single dose regimens (Arbiv et al., 2025). Our *in vivo* murine data demonstrate good tolerability over an 8-week treatment period without adverse clinical signs. This is consistent with high-dose rifampicin and moxifloxacin studies that have evaluated adverse effects and human clinical safety.

Histopathological analysis of lung tissues from mice treated with high-dose rifamycins and moxifloxacin revealed less inflammation than the HRZE-treated group, which indicates that HD-RIF + HZM and HD-RPT + HZM regimens improved bacterial reduction and led to less inflammatory damage. A previous study demonstrated that effective treatment regimens reduce overall pulmonary pathology, correlating with reduced lung bacterial burden (Budak et al., 2023). The reduction in iBALT (inducible Bronchus-Associated Lymphoid Tissue) formation in the high-dose rifamycin groups in our study indicates that these regimens are more effective at reducing bacterial burden and preventing the formation of chronic inflammatory structures compared to the HRZE-treated group. These findings suggest that these regimens reduce bacterial load and limit the immune-mediated damage caused by TB infection. The one minor exception was that rare cholesterol clefts were observed in all HRZE-treated mice; cholesterol clefts were inconsistently observed in HD-RIF + HZE, HD-RIF + HZM, and HD-RPT + HZM-treated mice. Cholesterol cleft formation occurs in response to cellular degeneration, often associated with necrosis. As such, the active cellular degeneration observed in the HRZE treatment group may correlate with this group having detectable lung CFU even after 2-months of treatment. These findings are significant as they provide additional evidence that high-dose rifamycin-based regimens could reduce the long-term sequelae of TB, particularly the lung damage associated with persistent infection and granuloma formation. These findings offer a preclinical rationale that reinforces the adoption of HRZM as a standard regimen for drug-susceptible TB, particularly for treatment-shortening strategies now validated in the clinic (Dorman et al., 2021).

## Conclusion

This back-translational study corroborates earlier findings and demonstrates that combining high-dose rifamycins with moxifloxacin can further enhance bacterial clearance, providing strong evidence for the need to re-evaluate the dosing strategies of current TB regimens. The combined use of high-dose rifamycins and moxifloxacin represents a promising strategy for the development of shorter, more effective TB regimens, particularly for drug-susceptible TB and cases at risk of relapse. It is important to emphasize that the high doses of rifamycins and moxifloxacin used in our murine model may not directly translate to clinically acceptable doses for a minority of TB patients. Future research should focus on assessing the long-term effects of these regimens, particularly in the context of relapse prevention. It has been emphasized that relapse remains one of the significant challenges in TB therapy, and studies incorporating relapse arms will be critical for understanding the true potential of these regimens in clinical practice. Additionally, dose optimization and fractionation studies could help identify the most effective dosing schedules that balance efficacy with tolerability.

## Data Availability

The original contributions presented in the study are included in the article/Supplementary Material, further inquiries can be directed to the corresponding author.

## Supplementary material

SUPPLEMENTARY FIGURE S1 Body weight trajectories in untreated and treated mice. Body weights of individual mice were recorded over the course of the study to monitor general health and potential treatment-related toxicity. Mice were treated with various regimens including HRZE, HD-RIF + HZE, HD-RIF + HZM, HD-RPT + HZE, and HD-RPT + HZM. Weights were measured at regular intervals throughout the 54-day experimental period. Weights observed in any treatment group relative to baseline, indicates treatments were well tolerated.
