# Correction: Evaluating the efficacy of HRZE-based regimens in a high-burden murine model: a back-translational assessment of rifamycins and moxifloxacin substitutions in tuberculosis treatment

**DOI:** 10.3389/fphar.2025.1733648

**Published:** 2025-11-18

**Authors:** Jason E. Cummings, Lisa K. Woolhiser, Vincent Guglielmi, Machenzie Wernsman, Ashley Romano, Samantha Pauly, John T. Belisle, Nicholas D. Walter, Gregory T. Robertson, Richard A. Slayden

**Affiliations:** 1 Mycobacteria Research Laboratories, Microbiology, Immunology and Pathology, Colorado State University, Fort Collins, CO, United States; 2 Consortium for Applied Microbial Metrics, Aurora, CO, United States; 3 Division of Pulmonary Sciences and Critical Care Medicine, University of Colorado Anschutz Medical Campus, Aurora, CO, United States; 4 Rocky Mountain Regional VA Medical Center, Aurora, CO, United States

**Keywords:** high-burden aerosol BALB/c model, high burden, tuberculosis, mycobacteria, drug discovery

There was a mistake in Figure 1 as published. **Figure S1** was published in place of the true Figure 1. The corrected Figure 1 appears below.

**FIGURE 1:**
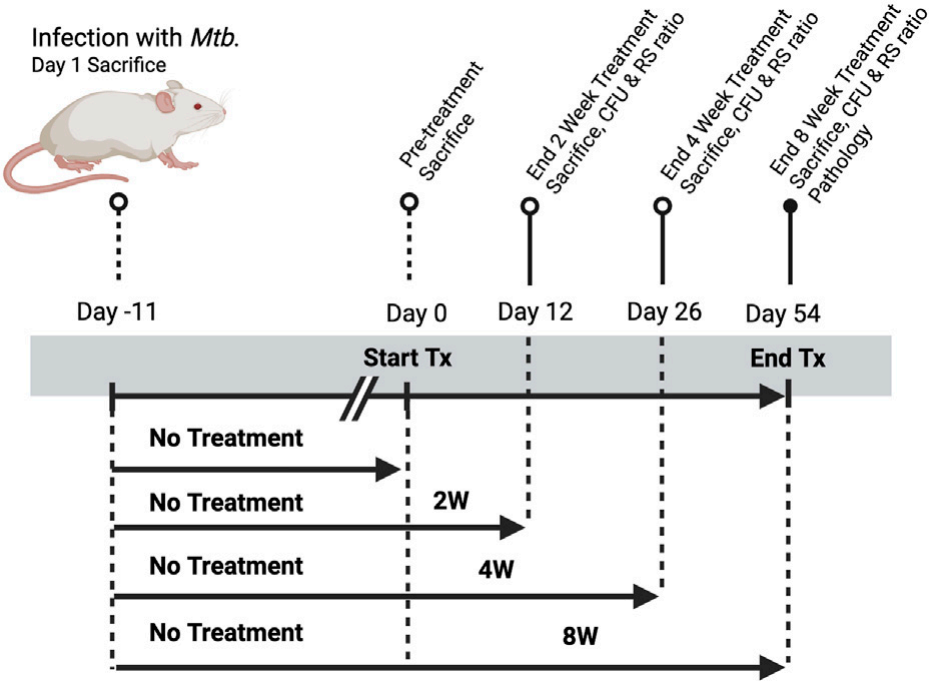
Study design for evaluating treatment duration-dependent responses to standard HRZE therapy in a murine model of *Mycobacterium tuberculosis* infection. Mice were aerosol-infected with *M. tuberculosis* on Day−11 and one group was sacrificed at Day 1 post-infection to establish baseline lung bacterial burden. All remaining animals remained untreated until Day 0, at which point treatment (Tx) was initiated. Groups received treatment for 2, 4, or 8 weeks, with corresponding sacrifices on Days 12, 26, and 54 to assess treatment efficacy. Endpoints included colony-forming unit (CFU) enumeration and determination of RS Ratio. Day 54 mice were also evaluated for histopathological changes and PK analysis. A parallel untreated control group was maintained through the full 8-week period.

Supplemental material **Figure S1** was omitted. The file has now been published.

The original article has been updated.

