# Supplementary material for: Evaluating the efficacy of HRZE-based regimens in a high-burden murine model: a back-translational assessment of rifamycins and moxifloxacin substitutions in tuberculosis treatment
Source: Front Pharmacol. 2025 Sep 15;16:1667592. doi: 10.3389/fphar.2025.1667592 (PMC12477428; doi:10.3389/fphar.2025.1667592)
Supplement: Supplementary file 1 [file Table1.docx]

**Table S1. Lung CFU Statistics**

|  | **Day 12** | Untreated Control | HRZE | HD-RIF | HD-RIF+HZE | HD-RIF+HZM | HD-RPT | HD-RPT+HZE | HD-RPT+HZM |
| --- | --- | --- | --- | --- | --- | --- | --- | --- | --- |
|  | Mean Lung Log_10_ CFU | 6.59 | 5.11 | 4.86 | 4.64 | 3.7 | 4.33 | 4.15 | 3.91 |
|  | SEM Lung Log_10_ CFU | 0.08 | 0.12 | 0.16 | 0.11 | 0.06 | 0.21 | 0.11 | 0.14 |
| Compared to Untreated Control | Log10 CFU drop | N/A | 1.48 | 1.73 | 1.95 | 2.89 | 2.26 | 2.44 | 2.68 |
|  | ANOVA | N/A | <.001 | <.001 | <.001 | <.001 | <.001 | <.001 | <.001 |
|  | Cohen's d | N/A | 4.59 | 4.25 | 6.44 | 12.90 | 4.45 | 7.96 | 7.60 |
|  | **Day 26** | Untreated Control | HRZE | HD-RIF | HD-RIF+HZE | HD-RIF+HZM | HD-RPT | HD-RPT+HZE | HD-RPT+HZM |
|  | Mean Lung Log_10_ CFU | 6.59 | 3.58 | 2.68 | 2.83 | 1.88 | 2.53 | 2.26 | 1.8 |
|  | SEM Lung Log_10_ CFU | 0.08 | 0.07 | 0.09 | 0.05 | 0.05 | 0.09 | 0.08 | 0.15 |
| Compared to Untreated Control | Log10 CFU drop | N/A | 3.01 | 3.91 | 3.76 | 4.71 | 4.06 | 4.33 | 4.79 |
|  | ANOVA | N/A | <.001 | <.001 | <.001 | <.001 | <.001 | <.001 | <.001 |
|  | Cohen's d | N/A | 12.50 | 14.65 | 17.62 | 22.07 | 15.33 | 17.25 | 13.56 |
|  | **Day 54** | Untreated Control | HRZE | HD-RIF | HD-RIF+HZE | HD-RIF+HZM | HD-RPT | HD-RPT+HZE | HD-RPT+HZM |
|  | Mean Lung Log_10_ CFU | 6.59 | 1.48 | 0.83 | 0.83 | 0.83 | 0.83 | 0.83 | 0.83 |
|  | SEM Lung Log_10_ CFU | 0.08 | 0.10 | 0.00 | 0.00 | 0.00 | 0.00 | 0.00 | 0.00 |
| Compared to Untreated Control | Log10 CFU drop | N/A | 5.11 | 5.76 | 5.76 | 5.76 | 5.76 | 5.76 | 5.76 |
|  | ANOVA | N/A | <.001 | <.001 | <.001 | <.001 | <.001 | <.001 | <.001 |
|  | Cohen's d | N/A | 17.88 | 32.26 | 32.32 | 32.32 | 31.35 | 31.35 | 32.32 |
