# Supplementary figures and images for: Evaluating the efficacy of HRZE-based regimens in a high-burden murine model: a back-translational assessment of rifamycins and moxifloxacin substitutions in tuberculosis treatment
Source: Front Pharmacol. 2025 Sep 15;16:1667592. doi: 10.3389/fphar.2025.1667592 (PMC12477428; doi:10.3389/fphar.2025.1667592)

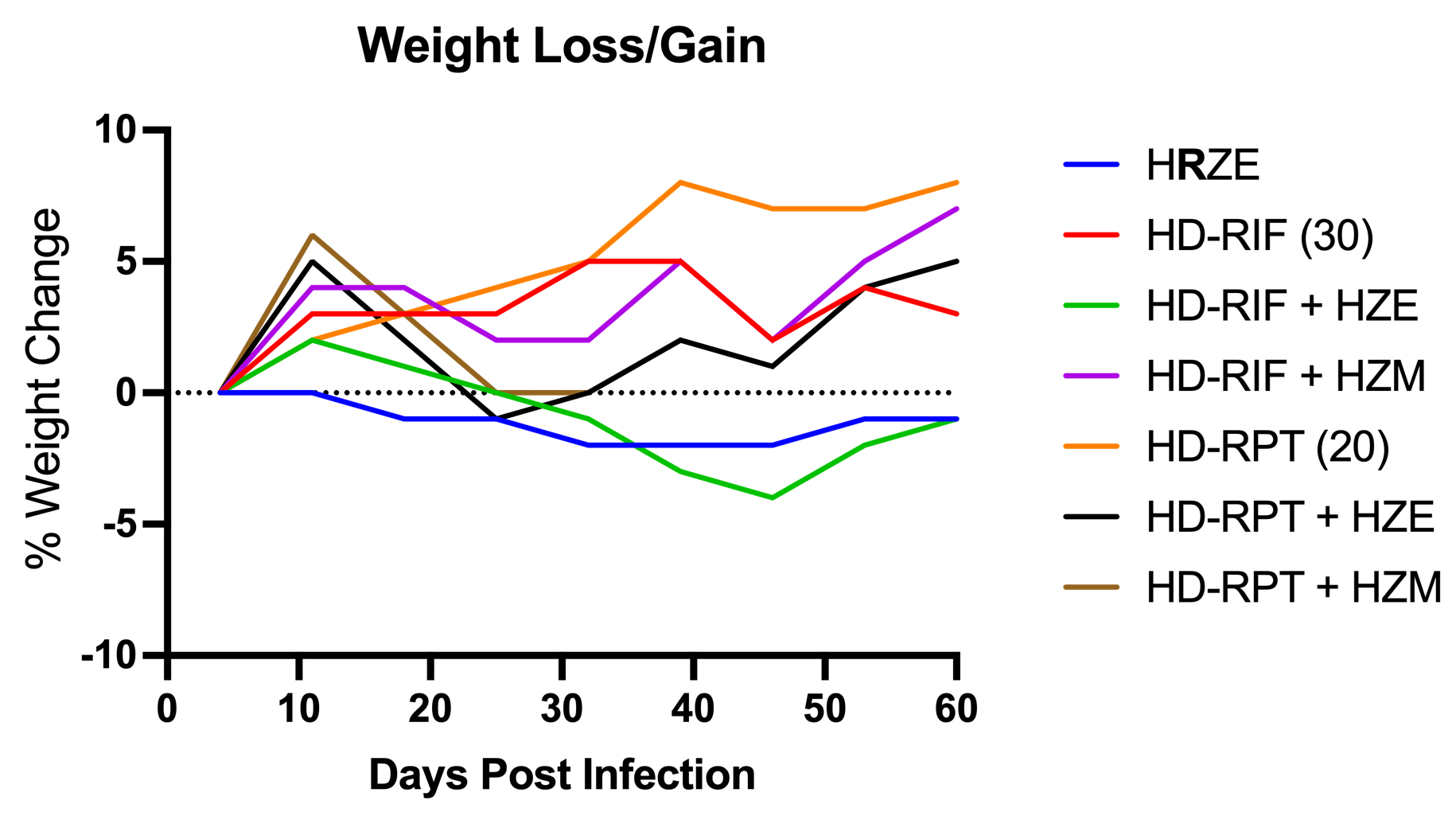

Supplement: Supplementary file 2 [file Image1.jpeg]
