# Supplementary material for: Evaluating the efficacy of HRZE-based regimens in a high-burden murine model: a back-translational assessment of rifamycins and moxifloxacin substitutions in tuberculosis treatment
Source: Front Pharmacol. 2025 Sep 15;16:1667592. doi: 10.3389/fphar.2025.1667592 (PMC12477428; doi:10.3389/fphar.2025.1667592)
Supplement: Supplementary file 3 [file Table2.docx]

**Table S2. RS Ratio Statistics**

|  | **Day 12** | Untreated Control | HRZE | HD-RIF | HD-RIF+HZE | HD-RIF+HZM | HD-RPT | HD-RPT+HZE | HD-RPT+HZM |
| --- | --- | --- | --- | --- | --- | --- | --- | --- | --- |
|  | Mean RS Ratio | 222.00 | 16.00 | 11.70 | 9.46 | 6.42 | 12.10 | 8.10 | 4.99 |
|  | SEM RS Ratio | 8.05 | 0.94 | 0.47 | 0.69 | 0.64 | 0.84 | 0.65 | 0.45 |
| Compared to Untreated Control | RS Ratio drop | N/A | 206 | 210.3 | 212.54 | 215.58 | 209.9 | 213.9 | 217.01 |
|  | ANOVA | N/A | <.001 | <.001 | <.001 | <.001 | <.001 | <.001 | <.001 |
|  | **Day 26** | Untreated Control | HRZE | HD-RIF | HD-RIF+HZE | HD-RIF+HZM | HD-RPT | HD-RPT+HZE | HD-RPT+HZM |
|  | Mean RS Ratio | 222.00 | 14.30 | 11.60 | 7.47 | 5.13 | 15.00 | 6.95 | 5.05 |
|  | SEM RS Ratio | 8.05 | 1.04 | 0.62 | 0.35 | 0.34 | 0.71 | 0.44 | 0.30 |
| Compared to Untreated Control | RS Ratio drop | N/A | 207.7 | 210.4 | 214.53 | 216.87 | 207 | 215.05 | 216.95 |
|  | ANOVA | N/A | <.001 | <.001 | <.001 | <.001 | <.001 | <.001 | <.001 |
|  | **Day 54** | Untreated Control | HRZE | HD-RIF | HD-RIF+HZE | HD-RIF+HZM | HD-RPT | HD-RPT+HZE | HD-RPT+HZM |
|  | Mean RS Ratio | 222.00 | 6.66 | 9.23 | 2.16 | 2.06 | 7.32 | 2.19 | 1.76 |
|  | SEM RS Ratio | 8.05 | 0.62 | 0.98 | 0.20 | 0.27 | 0.73 | 0.24 | 0.15 |
| Compared to Untreated Control | RS Ratio drop | N/A | 215.34 | 212.77 | 219.84 | 219.94 | 214.68 | 219.81 | 220.24 |
|  | ANOVA | N/A | <.001 | <.001 | <.001 | <.001 | <.001 | <.001 | <.001 |
