# Supplementary material for: Evaluating the efficacy of HRZE-based regimens in a high-burden murine model: a back-translational assessment of rifamycins and moxifloxacin substitutions in tuberculosis treatment
Source: Front Pharmacol. 2025 Sep 15;16:1667592. doi: 10.3389/fphar.2025.1667592 (PMC12477428; doi:10.3389/fphar.2025.1667592)
Supplement: Supplementary file 4 [file Table3.docx]

**Table S3. Plasma Drug Concentrations (ng/ml)**

|  |  | RIF |  | RPT |  | PZA |  |
| --- | --- | --- | --- | --- | --- | --- | --- |
| Treatment |  | Cmax | Stdev | Cmax | Stdev | Cmax | Stdev |
| HRZE | 1 hr | 11356.25 | 3.54 | x | x | 77604.80 | 2.55 |
|  | 24 hr | 1073.46 | 0.15 | x | x | 901.94 | 0.14 |
| HD-RIF | 1 hr | 29564.91 | 3.10 | x | x | x | x |
|  | 24 hr | 2414.05 | 0.28 | x | x | x | x |
| HD-RIF+HZE | 1 hr | 29727.72 | 8.60 | x | x | 64400.18 | 9.14 |
|  | 24 hr | 5402.54 | 4.00 | x | x | 602.42 | 0.22 |
| HD-RIF+HZM | 1 hr | 27404.31 | 2.85 | x | x | 68653.26 | 19.57 |
|  | 24 hr | 4010.40 | 0.10 | x | x | 484.38 | 0.04 |
| HD-RPT | 1 hr | x | x | 34796.32 | 5.38 | x | x |
|  | 24 hr | x | x | 22571.02 | 1.79 | x | x |
| HD-RPT+HZE | 1 hr | x | x | 37054.49 | 3.32 | 73570.57 | 1.07 |
|  | 24 hr | x | x | 29025.93 | 1.58 | 530.02 | 0.07 |
| HD-RPT+HZM | 1 hr | x | x | 42220.03 | 4.70 | 86271.91 | 17.41 |
|  | 24 hr | x | x | 37934.91 | 1.36 | 128.07 | 0.05 |
|  |  | INH |  | EMB |  | MOX |  |
| Treatment |  | Cmax | Stdev | Cmax | Stdev | Cmax | Stdev |
| HRZE | 1 hr | 3745.44 | 0.04 | 2125.10 | 0.82 | x | x |
|  | 24 hr | 2413.58 | 0.00 | not detectable |  | x | x |
| HD-RIF | 1 hr | x | x | x | x | x | x |
|  | 24 hr | x | x | x | x | x | x |
| HD-RIF+HZE | 1 hr | 3350.57 | 0.15 | 393.63 | 0.47 | x | x |
|  | 24 hr | 2408.82 | 0.00 | not detectable |  | x | x |
| HD-RIF+HZM | 1 hr | 3019.03 | 0.28 | x | x | 816.27 | 0.62 |
|  | 24 hr | 2408.62 | 0.00 | x | x | not detectable |  |
| HD-RPT | 1 hr | x | x | x | x | x | x |
|  | 24 hr | x | x | x | x | x | x |
| HD-RPT+HZE | 1 hr | 3582.30 | 0.26 | 3165.65 | 2.56 | x | x |
|  | 24 hr | 2662.08 | 0.29 | not detectable |  | x | x |
| HD-RPT+HZM | 1 hr | 3754.56 | 0.14 | x | x | 1285.99 | 0.62 |
|  | 24 hr | 2914.09 | 0.00 | x | x | not detectable |  |
